# Foxa1 Reduces Lipid Accumulation in Human Hepatocytes and Is Down-Regulated in Nonalcoholic Fatty Liver

**DOI:** 10.1371/journal.pone.0030014

**Published:** 2012-01-06

**Authors:** Marta Moya, Marta Benet, Carla Guzmán, Laia Tolosa, Carmelo García-Monzón, Eugenia Pareja, José Vicente Castell, Ramiro Jover

**Affiliations:** 1 Experimental Hepatology Unit, University Hospital La Fe, Valencia, Spain; 2 Surgery and Liver Transplantation Unit, University Hospital La Fe, Valencia, Spain; 3 CIBERehd, Centro de Investigación Biomédica en Red de Enfermedades Hepáticas y Digestivas, Barcelona, Spain; 4 Liver Research Unit, Instituto de Investigación Sanitaria Princesa, University Hospital Santa Cristina, Madrid, Spain; 5 Department of Biochemistry and Molecular Biology, University of Valencia, Valencia, Spain; Université Joseph Fourier, France

## Abstract

Triglyceride accumulation in nonalcoholic fatty liver (NAFL) results from unbalanced lipid metabolism which, in the liver, is controlled by several transcription factors. The Foxa subfamily of winged helix/forkhead box (Fox) transcription factors comprises three members which play important roles in controlling both metabolism and homeostasis through the regulation of multiple target genes in the liver, pancreas and adipose tissue. In the mouse liver, Foxa2 is repressed by insulin and mediates fasting responses. Unlike Foxa2 however, the role of Foxa1 in the liver has not yet been investigated in detail. In this study, we evaluate the role of Foxa1 in two human liver cell models, primary cultured hepatocytes and HepG2 cells, by adenoviral infection. Moreover, human and rat livers were analyzed to determine Foxa1 regulation in NAFL. Results demonstrate that Foxa1 is a potent inhibitor of hepatic triglyceride synthesis, accumulation and secretion by repressing the expression of multiple target genes of these pathways (e.g., GPAM, DGAT2, MTP, APOB). Moreover, Foxa1 represses the fatty acid transporter protein FATP2 and lowers fatty acid uptake. Foxa1 also increases the breakdown of fatty acids by inducing peroxisomal fatty acid β-oxidation and ketone body synthesis. Finally, Foxa1 is able to largely up-regulate UCP1, thereby dissipating energy and consistently decreasing the mitochondria membrane potential. We also report that human and rat NAFL have a reduced Foxa1 expression, possibly through a protein kinase C-dependent pathway. We conclude that Foxa1 is an antisteatotic factor that coordinately tunes several lipid metabolic pathways to block triglyceride accumulation in hepatocytes. However, Foxa1 is down-regulated in human and rat NAFL and, therefore, increasing Foxa1 levels could protect from steatosis. Altogether, we suggest that Foxa1 could be a novel therapeutic target for NAFL disease and insulin resistance.

## Introduction

The Foxa subfamily of winged helix/forkhead box (Fox) transcription factors comprises three members (Foxa1, Foxa2 and Foxa3) which play important roles in early mammalian development and organogenesis. In postnatal life, the Foxa transcription factors are also highly relevant because they play key roles in controlling metabolism and homeostasis through the regulation of multiple target genes in the liver, pancreas and adipose tissue [1].

In the livers of adult mice, Foxa2 activity mediates fasting responses, including fatty acid (FA) oxidation, ketogenesis, gluconeogenesis and increased lipoprotein secretion, by activating the expression of key genes of these pathways [2], [3], [4]. Foxa2 also performs a crucial role in hepatic bile acid homeostasis and in the prevention of cholestatic liver injury [5]. In the postprandial state when insulin levels rise, Foxa2 is phosphorylated at Thr 156 through phosphatidylinositol 3-kinase/Akt signaling [6]. This results in nuclear exclusion of Foxa2 [3] and inhibition of its activity [7]. In hyperinsulinemic/obese mice, Foxa2 is permanently inactive, which contributes to the development of hepatic steatosis and insulin resistance [3].

Foxa1 and Foxa3 are not phosphorylated by insulin-signaling cascades [6]. Nevertheless, Foxa3 also plays an important role in liver metabolism as it regulates glucose homeostasis during a prolonged fast through the maintenance of GLUT2 and gluconeogenic gene expression [8].

In contrast to Foxa2 and Foxa3, the role of Foxa1 in liver metabolism seems to be less critical. Foxa1 null mice develop a phenotype characterized by progressive starvation, persistent hypoglycemia, hypotriglyceridemia and neonatal mortality [9]. This phenotype, however, does not arise from liver dysfunction, but develops because Foxa1 mediates glucagon gene expression and insulin secretion in pancreatic cells [9], [10]. The lack of a significant hepatic phenotype in Foxa1 null mice may be explained by Foxa2 and Foxa3 being able to compensate for the loss of Foxa1 without gross alterations in gene expression of most Foxa target genes. Similarly, mild hepatic phenotypes have been found in Foxa2 and Foxa3 null mice [11], [12].

To overcome this functional redundancy, we investigated the role of Foxa1 in human hepatocytes and hepatoma cells by gain-of-function experiments and found that Foxa1 is a potent inhibitor of hepatic triglyceride (TG) synthesis and secretion. Moreover, Foxa1 inhibits FA uptake and stimulates FA oxidation, ketone body (KB) synthesis and energy dissipation in the mitochondria, which indicates that Foxa1 can coordinate several transcriptional responses to lower intracellular lipid levels in hepatocytes. We also found that human and rat nonalcoholic fatty liver (NAFL) has a reduced Foxa1 expression, which could promote the incorporation of excessive circulating lipids into hepatocytes. Consistently, when Foxa1 levels are high, steatosis significantly lowers.

## Materials and Methods

### Ethics Statements

Studies involving human samples or patients were performed in agreement with the Declaration of Helsinki and with local and national laws. The Human Ethics Committee of the respective hospitals (Hospital La Fe Valencia and Hospital Santa Cristina Madrid) approved the study procedures, and written informed consent was obtained for all participants before inclusion.

Studies with laboratory animals followed the guidelines of, and were approved by, the Animal Research Ethical Committee of the University Hospital La Fe, Valencia (Approval ID: IIS-2010/0023).

### Patients and human liver bank

Part of this study comprised 17 nondiabetic patients with NAFL who showed biopsy-proven steatosis (grade 1 or 2) without necroinflammation or fibrosis; and 17 patients with asymptomatic cholelithiasis from whom a liver biopsy was taken during programmed laparoscopic cholecystectomy (normal liver was confirmed). None of the 34 patients drank more than 20 g of alcohol per day and none showed evidence of viral infections.

The Human Liver Bank of the Hospital La Fe-CIBERehd (SPAIN) is a collection of donor livers which are primarily assigned to liver transplantation but, due to failings in some of the inclusion criteria (e.g. steatosis) they are donated to research.

### Animals

Adult male OFA rats from Charles River (Barcelona, Spain) were housed two per cage and acclimatized to laboratory conditions for 7 days (12-hour light-dark cycle, 21–25°C, 30–70% humidity, woodchip bedding). Rats were fed *ad libitum* for 7 weeks with either a methionine- and choline-deficient (MCD) diet or standard isocaloric chow (#A02082002B and #A02082003B, Research Diets INC. New Brunswick, NJ). Livers were perfused and frozen in liquid N_2_.

### Isolation and culture of human hepatocytes and cell lines

Human hepatocytes were isolated from nonsteatotic biopsies (1–4 g) using a two-step perfusion technique [13]. Liver samples were obtained in conformity with the rules of the Hospital's Ethics Committee. None of the donors (7 men and 6 women aged between 22 and 74) were regular consumers of alcohol or of other drugs, and were not suspected of harboring any infectious disease. Cells were cultured in serum-free Williams/Ham's F-12 (1/1, v/v) medium (Gibco BRL, Invitrogen, Barcelona, Spain), supplemented with 5 mM glucose, 0.2% bovine serum albumin, 0.01 µM insulin and 0.01 µM dexamethasone.

HepG2 cells (ATCC HB-8065, Rockville, MD) were cultured in Ham's F-12/Leibovitz L-15 (1∶1, v/v) medium (Gibco BRL, Invitrogen, Barcelona, Spain), supplemented with 7% newborn calf serum and 2 mM L-Glutamine. When incubated with FA, HepG2 were cultured in the same serum-free medium as human hepatocytes. In addition, culture media were supplemented with 50 U/ml penicillin and 50 µg/ml streptomycin.

To induce fat accumulation, cultured cells were exposed to oleate and palmitate (2∶1 ratio) (Sigma-Aldrich, Madrid, Spain), in serum-free culture medium containing 1% FA-free BSA (Sigma-Aldrich, Madrid, Spain). To assess the effect of PKC activation, cells were exposed to 160 nM Phorbol 12-Myristate 13-Acetate (PMA, Sigma-Aldrich, Madrid, Spain). Cells were also treated with 200 nM Gö-6976 or 1 µM Ro-318220 as PKC inhibitors (Sigma-Aldrich, Madrid, Spain). When both PKC activator (PMA) and inhibitors (Gö-6976 or Ro-318220) were studied, inhibitors were added to cultures 1 h before the activator.

### Development of an adenoviral vector encoding human Foxa1

The shuttle plasmid pAC-Foxa1 was constructed by ligation of Foxa1 cDNA (kindly provided by Dr R Bort, UHE-La Fe, Valencia, Spain) pre-digested with EcoRI-BamHI into the adenoviral vector pAC/CMVpLpA [14]. This plasmid and the pJM17 containing the dl309 adenovirus-5 genome, were co-transfected into 293 with FuGENE HD Transfection Reagent (Roche Applied Sciences, Barcelona, Spain). The homologous recombination between these two plasmids resulted in a replication defective virus (Ad-Foxa1), which was plaque-cloned, amplified and purified, with the Vivapure AdenoPACK TM100 Kit from Sartorius (Goettingen, Germany), into a high-concentration stock of 2.4×10^7^ plaque-forming units (PFU)/µl [14]. Human hepatocytes and HepG2 cells were infected with Ad-Foxa1 or an insertless adenoviral vector (Ad-Control) for 24 h at a multiplicity of infection (MOI) ranging from 1 to 36 PFU/cell. Thereafter, cells were washed and fresh medium was added. At 48 h post-transfection cells were harvested and analyzed. Adenoviral vectors achieved nearly 100% transfection efficiency as assessed by Ad-LacZ infection and β-galactosidase staining [14].

### Biochemical functional assays

The neutral lipid content of cultured cells was determined fluorometrically by Nile Red staining with a SpectraMAX GeminiXS Fluorometer as described elsewhere [13]. To correct potential variations in cell number, the DNA content was determined in the same well/plates by Hoechst 33258 staining (Sigma-Aldrich, Madrid, Spain).

The spectrophotometric assay of acyl-CoA oxidase (ACOX) activity was based on the determination of H_2_O_2_ production, which was coupled to the oxidation of 2,7-dichlorofluoroscein diacetate in a reaction catalyzed by exogenous peroxidase [15], [16]. Homogenates from infected HepG2 cells were sonicated in phosphate potassium buffer 60 mM (pH 7.4) and centrifuged to eliminate cell debris. The assay mixture (1 ml) containing 60 mM phosphate potassium buffer (pH 7.4), 0.02% Triton X100, 0.6 mg/ml BSA, 40 mM aminotriazole, 0.05 mM 2,7-Dichlorofluoroscein diacetate, 0.015 mM FAD and 50 µg horseradish peroxidase type II (all compounds from Sigma-Aldrich, Madrid, Spain) was incubated with 100 µg of homogenate in the dark for 5 minutes. After this time, 30 µM of palmitoyl-CoA was added or not to the mixture and absorbance at 502 nm was measured every 6 seconds during 2 minutes.

Free FA and ketone bodies (KB) were quantified in culture medium with the Free Fatty Acid Quantification Kit and the beta-Hydroxybutyrate (β-HB) Assay Kit, both from BioVision Research Products (Mountain View, CA).

Homogenates from human liver were extracted with a methanol-chloroform mixture and evaporated under nitrogen. Total lipid and TG contents were measured in the lipid residue using colorimetric kits from Spinreact (Gerona, Spain) based on the vanillin-phosphoric acid reaction (#1001270) and the GPO-POD enzymatic method (#1001311), respectively.

### Reverse transcription and real-time quantitative PCR

Total RNA was extracted with TRIzol Reagent from Invitrogen (Barcelona, Spain) and re-purified using the RNeasy Plus Mini Kit from Qiagen (Madrid, Spain). Total RNA (1 µg) was reverse transcribed as described [13] and real-time quantified using SYBR Green I Master (Roche Applied Sciences, Barcelona, Spain) and the appropriate primers (Table S1) in a LightCycler 480 Instrument. In parallel, two human housekeeping genes; porphobilinogen deaminase (PBGD) and glyceraldehyde 3-phosphate dehydrogenase (GAPDH); were analyzed for normalization. In each amplification, we included a reference calibrator cDNA made from 18 nonsteatotic human livers (Human Liver Bank, HLaFe-CIBERehd).

Relative mRNA levels were calculated with the LightCycler Relative Quantification Analysis software. Briefly, the efficiency of each PCR reaction was estimated from a serially diluted human liver cDNA curve. This curve allows to determine the PCR efficiency of each pair of primers. Based on these PCR efficiencies and the cycle thresholds (Ct), the software calculates the relative concentration of target and reference (PBGD or GAPDH) cDNAs and their ratio.

For absolute quantification, amplicons were first confirmed by agarose gel and purified with Wizard SV Gel and PCR Clean-up System (Promega Biotech Iberica, Madrid, Spain). Next, the amount of DNA was quantified with the Quant-it Picogreen dsDNA Assay Kit (Molecular Probes, Invitrogen, Barcelona, Spain), and properly diluted to be used as a reference standard curve to interpolate and estimate the absolute amount of target cDNA molecules in each sample.

### Microarray expression analysis

Total RNA was purified from quadruplicate experiments in HepG2 and cultured human hepatocytes infected with Ad-Foxa1 or control adenovirus (insertless Ad-pACC). mRNA expression profiles were analyzed with the GeneChip® Human Gene 1.0 ST Array (Affymetrix, Santa Clara, CA) as described [17]. Sample preparation and microarray hybridization and scanning were performed at the Gene Analysis Service (Central Research Unit (UCIM), Faculty of Medicine, University of Valencia). Microarray quality control assessment and data acquisition were performed with the GeneChip® Operating Software (Affymetrix). Normalization among the different microarray data files was performed by robust multiarray analysis. Next, we applied a conservative probe filtering (log_2_ expression value ≥5 in at least one sample). The number of probes selected was 13128 in human hepatocytes and 14833 in HepG2 cells from the original probe set of 32320. Differential mRNA expression was determined by using linear models and empirical Bayes paired moderated t statistics. False discovery rates were determined by following the Benjamini-Hochberg procedure. The data from this study have been prepared in compliance to MIAME guidelines and deposited in the Gene Expression Omnibus (GEO) database with reference numbers GSE30450 (human hepatocytes) and GSE30447 (HepG2).

### Protein samples and immunoblotting analysis

Liver and cell homogenates, and culture media, were centrifuged at 13000×g for 10 min at 4°C, and supernatants were quickly stored frozen in aliquots at −80°C. Tissue and cell culture samples were homogenized in T-PER and M-PER reagents from Pierce (Fisher Scientific, Madrid, Spain). Protein concentration was determined using the Protein Assay Kit from Bio-Rad Laboratories (Madrid, Spain). Proteins were resolved by 12% SDS-PAGE or 4–12% NuPage precast gels (Invitrogen, Barcelona, Spain), transferred to Immobilion membranes (Millipore Iberica, Madrid, Spain) and incubated with an anti-ApoB antibody (Rockland Immunochemicals, PA) or anti-Foxa1 antibodies (sc-6553 and sc-101058, Santa Cruz Biotechnology, CA). Blots were developed with horseradish peroxidase-labeled IgG using an ECL kit from Amersham Biosciences/GE Healthcare (Buckinghamshire, England). Equal loading was verified by Coomassie Brilliant Blue or Ponceau S staining.

### Olympus ScanR screening station

The high content screening imaging station Sca
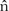
R from Olympus was used to analyze neutral lipid content, mitochondrial membrane potential (MMP) and FA uptake, by using the specific fluorescent probes BODIPY 493/503 (3.75 ng/ml), TMRM (75 ng/ml) and C1-BODIPY 500/510-C12 (0.4 µg/ml), respectively (Molecular Probes, Invitrogen, Barcelona, Spain). Cells were loaded with the appropriate combination of fluorescent probes for 15–30 min and the medium was replaced with a buffered medium (10 mM Hepes) for the analysis. The number of cells was calculated from the number of Hoechst 33342 (1.5 µg/ml) stained nuclei, and cell viability was determined by propidium iodide (1.5 µg/ml) exclusion, which allows to perform functional determinations only in the population of live cells. The 10X objective was used to collect images for the distinct fluorescence channels. Nine fields per well were acquired and analyzed by simultaneous quantification of the different probe fluorescence intensities in the predefined nuclear and cytoplasmic compartments.

### Statistical analysis

Statistical differences between group parameters were determined by the Student's t-test and the Mann-Whitney U test.

## Results

### mRNA levels of Foxa factors in human liver and cultured hepatic cells

**Figure 1:**
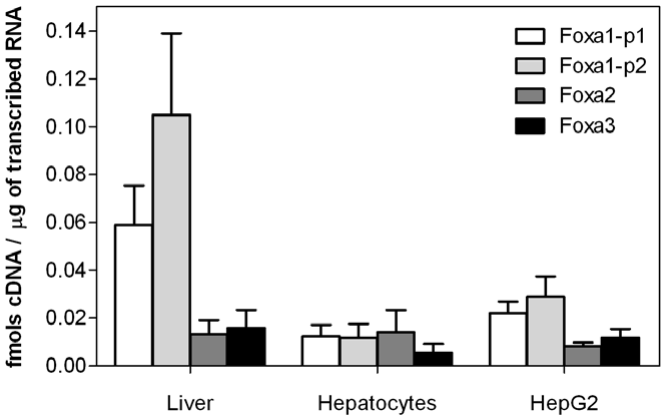
Foxa1 is more abundant than Foxa-2 and -3 in human liver, but its expression declines in liver cell cultures. Foxa-1, -2 and -3 mRNA concentrations were measured in human livers (n = 15), human hepatocyte cultures (48 h of culture, n = 8) and HepG2 cultures (n = 6) by real-time Q-RT-PCR. Foxa1 expression was assessed with two different primer pairs (Foxa1-p1 and Foxa1-p2). Absolute cDNA quantification was performed by interpolation in DNA standard curves with a known input concentration.

Previous studies have suggested that Foxa1 is less abundant in mouse liver than Foxa2 and Foxa3, despite the stronger gel mobility-shift activity of Foxa1 with crude liver extracts [18]. However, the relative expression level of the three Foxas in human liver has not yet been determined. We performed absolute mRNA quantification and found that, in the human liver, Foxa1 is more abundant than Foxa2 and 3 (Fig. 1). However in cultured human hepatocytes and HepG2 cells, Foxa1 mRNA substantially decreased (up to 15–40%), whereas Foxa2 and 3 mRNA levels remained similar to liver levels (Fig. 1).

### Over-expression of Foxa1 in cultured human liver cells

**Figure 2:**
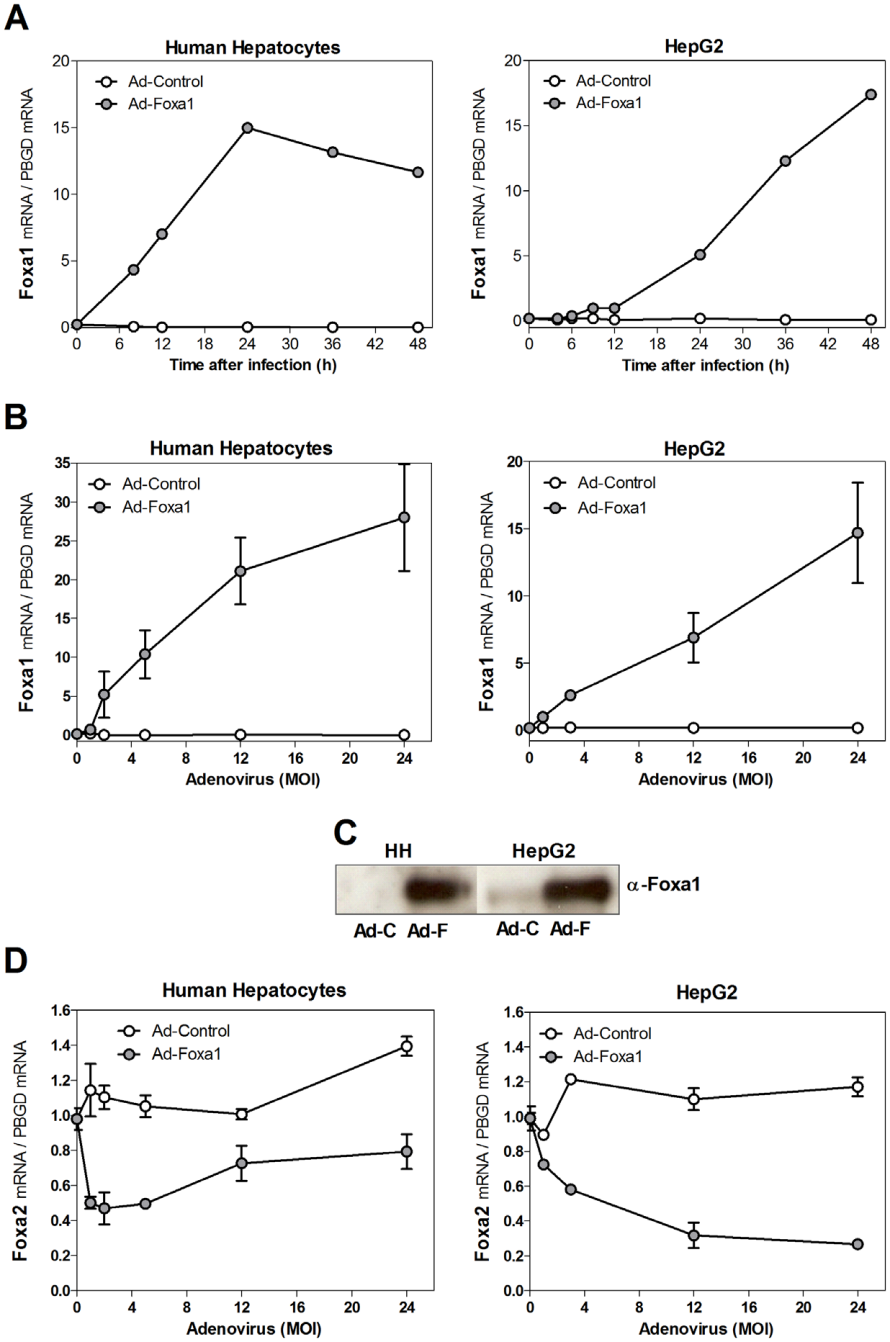
Adenovirus-mediated expression of Foxa1 in human liver cell cultures. HepG2 cells and cultured human hepatocytes were infected with Ad-Foxa1 or Ad-Control, and Foxa1 or Foxa2 mRNA levels were measured by Q-RT-PCR and normalized to the expression of the housekeeping PBGD mRNA. (**A**) Time-course analysis of Foxa1 mRNA after transduction of human hepatocytes and HepG2 cells with 5 and 24 MOI of adenoviral vectors, respectively. Dose-response analysis of Foxa1 (**B**) and Foxa2 (**D**) in human liver cultured cells after different MOI of adenovirus for 48 h. Data represent the mean±SD of 3 independent experiments. (**C**) Protein levels of Foxa1 determined by immunoblotting (sc-6553 antibody) in representative samples of human liver cultured cells after 12 (HH) or 24 (HepG2) MOI of adenovirus for 48 h (Ad-C: Ad-Control; Ad-F: Ad-Foxa1; HH: human hepatocytes). Equal loading was verified by Ponceau S staining.

To circumvent the down-regulation of Foxa1 in cultured cells and to overcome the functional redundancy among the three Foxa factors, we developed a recombinant adenovirus encoding human Foxa1. Transduction of cultured human hepatocytes and HepG2 cells with this adenovirus caused a significant, time- and dose-dependent increase in Foxa1 mRNA and protein levels (Fig. 2A, 2B and 2C). The infective doses of Ad-Foxa1, selected from the dose-response experiments, were set at 5–12 MOI for human hepatocytes and at 12–24 MOI for HepG2 cells.

Transduction of cultured cells with Ad-Foxa1 at the selected MOI led to an increase in Foxa1 mRNA ranging from 7 to 21-fold over the control level. This overexpression compensates for the down-regulation of Foxa1 in cultured cells but exceeds the averaged level in human liver.

We also evaluated the mRNA level of the other two Foxa family members, Foxa2 and Foxa3, in response to Ad-Foxa1, and found that human Foxa2 is significantly down-regulated by Foxa1, in both human hepatocytes and HepG2 cells (Fig. 2D). The mRNA level of Foxa3 was not substantially altered by Foxa1 (data not shown).

### Gene expression profile of Foxa1-transfected human hepatocytes and HepG2 cells

To determine the global impact of Foxa1 on human liver gene transcription, microarray expression analyses were performed in human hepatocytes and HepG2 cells transfected with Ad-Foxa1 or Ad-Control. We performed quadruplicated independent experiments and set the cut-off for the differentially expressed genes at a q value (False Discovery Rate) of ≤0.05 and a fold change of ±5/3.

The results from cultured human hepatocytes revealed 1140 differentially expressed genes, of which 434 were up-regulated by Foxa1 and 706 were down-regulated. The analyses in HepG2 cells showed 1061 differentially expressed genes, of which 674 were up-regulated by Foxa1 and 387 were down-regulated. The overlap of the gene lists from human hepatocytes and HepG2 cells in Venn diagrams was about 25%.

We next analyzed the overrepresented biochemical pathways in our differentially-expressed gene lists by using the ConsensusPathDB tool (Max Planck Institute for Molecular Genetics), which was set at a p-value cut-off of <0.01. We found more enriched pathway-based sets in the lists of down-regulated genes, and among them, several hepatic functions associated with FA, glycerophospholipid, lipoprotein and bile acid metabolism. These pathways were overrepresented in the gene lists of both human hepatocytes and HepG2 cells suggesting that Foxa1 is likely to play a significant regulatory role in lipid metabolism in the adult human liver.

Table S2 shows a list of lipid and carbohydrate metabolism genes with differential mRNA expression in human hepatocytes and HepG2 cells transfected with Ad-Foxa1 vs Ad-Control. Data suggest that Foxa1 represses FA uptake, TG and VLDL synthesis, FA synthesis/elongation/desaturation, and lipid droplet formation; whereas it induces FA catabolism and ketone body synthesis.

### Foxa1 inhibits the expression of genes involved in TG and VLDL synthesis

**Figure 3:**
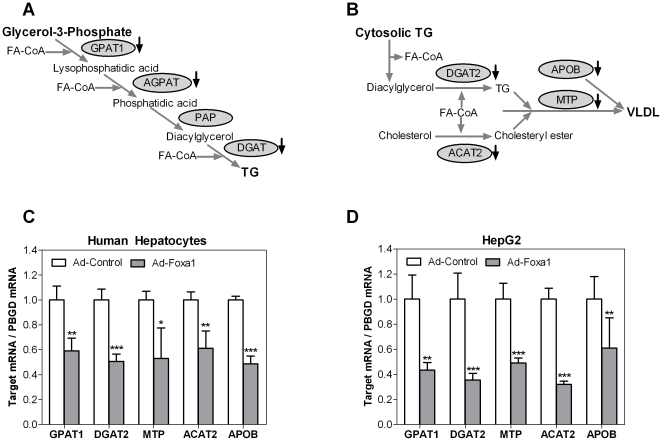
Foxa1 inhibits the expression of genes involved in TG and VLDL synthesis. Diagrams of TG (**A**) and VLDL (**B**) synthesis pathways in the liver. Human hepatocytes (**C**) and HepG2 cells (**D**) were infected with Ad-Foxa1 or Ad-Control (12 MOI for human hepatocytes and 24 MOI for HepG2) for 48 h, and the mRNA level of TAG and VLDL genes was measured by real-time Q-RT-PCR and normalized to the expression of the housekeeping PBGD mRNA. Data represent the mean±SD of 4–5 independent experiments. * p<0.05; ** p<0.01; *** p<0.001 in relation to Ad-Control infected cells. **GPAT**: glycerol-3 phosphate acyltransferase. **AGPAT**: 1-acylglycerol-3-phosphate-O-acyltransferase. **DGAT**: diacylglycerol acyltransferase. **MTP**: microsomal triglyceride transfer protein. **ACAT**: acyl coenzyme A: cholesterol acyltransferase. **APOB**: apolipoprotein B.

TG synthesis in the liver proceeds along a sequence involving four reactions in the liver: 3 esterification reactions and 1 hydrolysis reaction (Fig. 3A). Relative mRNA quantification by Q-RT-PCR confirmed that most of the genes involved in this pathway (GPAT1: glycerol-3 phosphate acyltransferase 1, AGPAT5 and 9: 1-acylglycerol-3-phosphate-O-acyltransferase 5 and 9, and DGAT2: diacylglycerol acyltransferase 2) were significantly repressed by Foxa1 in human hepatocytes (GPAT1 and DGAT2, Fig. 3C, and AGPAT5 and AGPAT9, data not shown). GPAT1 and DGAT2 were also down-regulated in Ad-Foxa1-transfected HepG2 cells (Fig. 3D).

The TG pathway is closely linked to the VLDL secretory pathway (Fig. 3B). The synthesis of VLDL is substrate-driven, and an elevated flux of free FA to the liver has hepatocytes primed to channel FA into TG and VLDL [19]. Conversely, reduced TG results in lower VLDL synthesis rates. Indeed, most of the genes involved in VLDL synthesis (DGAT2, MTP: microsomal triglyceride transfer protein, ACAT2: acyl coenzyme A: cholesterol acyltransferase 2, and APOB: apolipoprotein B) were also significantly down-regulated by Foxa1 in hepatocytes (Fig. 3C) and in HepG2 cells (Fig. 3D). Altogether, our results suggest that Foxa1 can coordinately inhibit the key human genes involved in TG and VLDL synthesis in the liver, which should result in the blockage of TG accumulation and lipoprotein secretion by hepatocytes.

### Foxa1 reduces steatosis and ApoB100 secretion in cultured human liver cells

**Figure 4:**
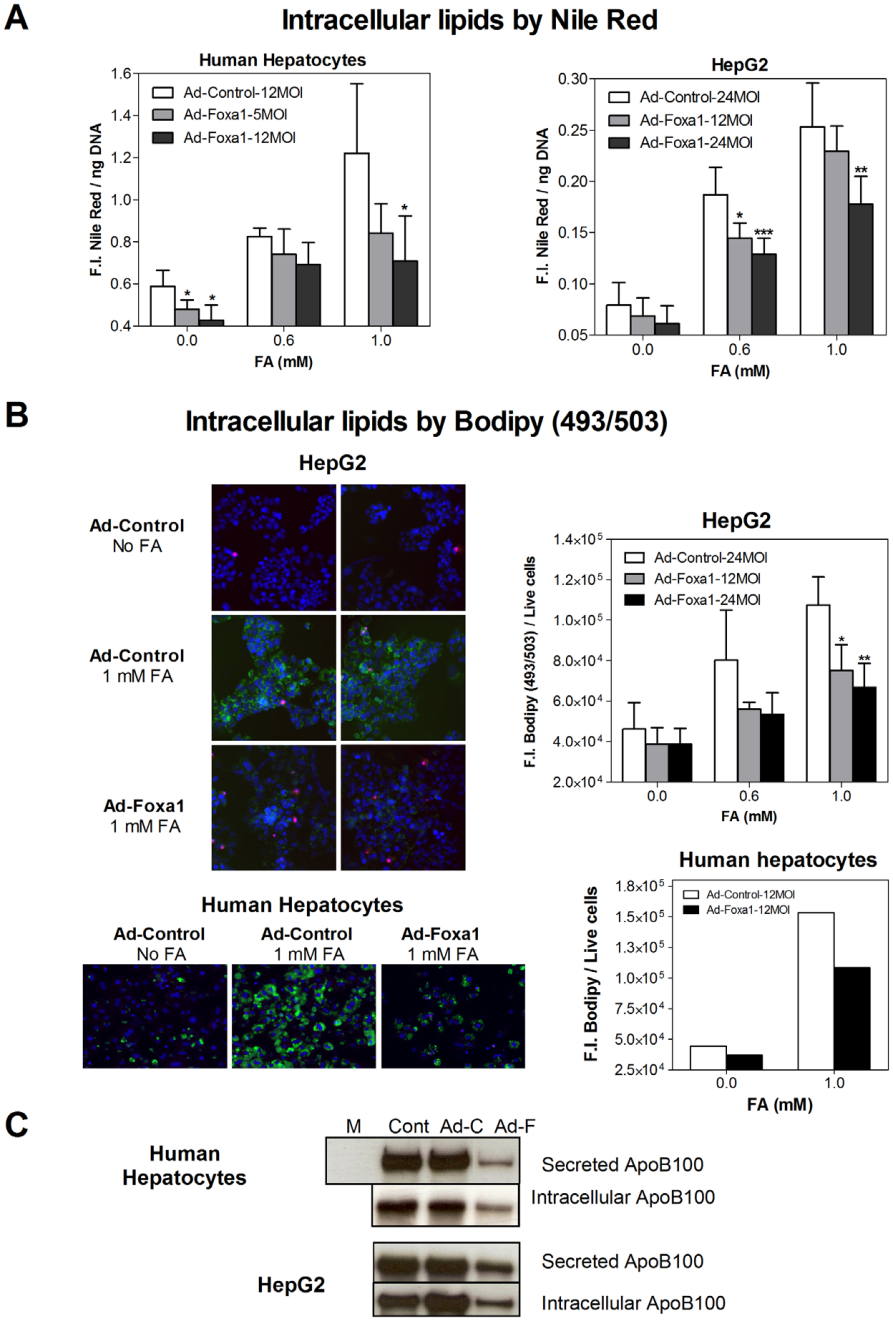
Foxa1 reduces lipid accumulation and ApoB100 secretion in human liver cells. (**A**) Cultured human hepatocytes and HepG2 cells were transduced with Ad-Foxa1 or Ad-Control for 24 h and then exposed to FA (oleate: palmitate, 2∶1). Twenty-four hours later, accumulated lipids were quantified by Nile Red fluorescence. Data represent the mean±SD of 4–5 independent experiments. * p<0.05; ** p<0.01; *** p<0.001. (**B**) Left panels: Representative microphotographs of living HepG2 cells and human cultured hepatocytes showing cytoplasmic neutral lipids stained with BODIPY 493/503 (green), nuclei stained with Hoechst 33342 (blue) and death cell nuclei stained with propidium iodide (pink). Right panels: Quantification of the BODIPY 493/503 fluorescent signal with the Olympus ScanR Screening Station in HepG2 cells (mean±SD, n = 4) and in a representative human hepatocyte culture. (**C**) Secreted (culture medium) and intracellular ApoB100 protein was analyzed by immunoblotting at 48h after infection with adenoviral vectors. (M, fresh culture medium; Cont, non-infected controls; Ad-C, Ad-Control; Ad-F, Ad-Foxa1).

When primary human hepatocytes and HepG2 cells are cultured in the presence of oleate and palmitate (2∶1 ratio, 1 mM, 24 h), they accumulate intracellular lipids without cytotoxic or apoptotic side effects [20]. The level of steatosis attained is similar to that measured in human NAFL [20]. To test the effect of Foxa1 on lipid accumulation and storage, cultures were first transduced with Ad-Foxa1 and then exposed to FA. The quantification of accumulated lipids by Nile Red fluorescence demonstrated that Foxa1 significantly reduces steatosis in human hepatocytes and HepG2 cells (Fig. 4A).

In order to confirm these results, we used an Olympus Sca
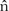
R Screening Station for automated analysis of cultured living cells and the BODIPY 493/503 probe to stain intracellular neutral lipids. Microphotographs in Fig. 4B show an increase in green fluorescence by accumulated neutral lipids when cells were loaded with 1mM FA (24 h). However, transduction with Ad-Foxa1 largely reduced this fluorescent signal in both HepG2 cells and human hepatocytes. The quantification of the fluorescence intensities in living cells once again revealed a significant inhibition of lipid accumulation by Foxa1 (Fig. 4B), which reinforces the idea that this transcription factor can reduce steatosis in human liver cells.

We also investigated the functional relevance of the inhibition of VLDL genes by Foxa1. Immunoblotting analysis showed a strong reduction in both intracellular and secreted (culture medium) ApoB100 protein, when cells were infected with Ad-Foxa1 (Fig. 4C). These results were observed in both human hepatocytes and HepG2 cells, and strongly suggest that VLDL synthesis is also effectively inhibited by Foxa1.

### Foxa1 reduces FA transport, increases FA catabolism and uncouples mitochondria

What is the fate of excessive extracellular FA in Foxa1-transfected cells? Two major possibilities may take place: less FA transport into hepatocytes or/and higher FA catabolism rates.

The most prominent and best characterized membrane proteins for FA uptake are: FABPpm, CD36/FAT, FATPs and caveolin [21]. FABPpm and FAT/CD36 are membrane-associated FA-binding proteins that facilitate FA translocation. However, FATPs have been suggested to be the real FA transporters and vectorial acylation has been proposed as the mechanism behind this [22].

**Figure 5:**
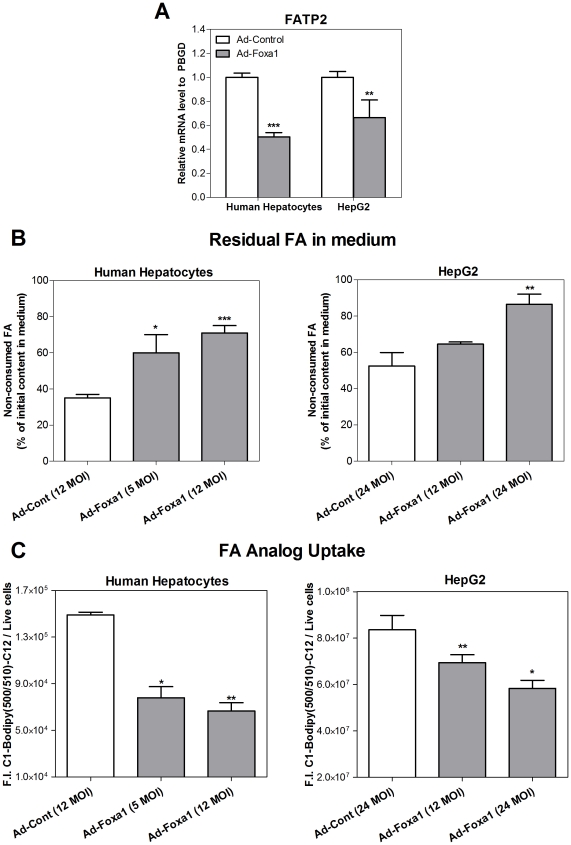
Foxa1 decreases FA uptake. (**A**) Cultured cells were infected with Ad-Foxa1 or Ad-Control for 48h, and the mRNA level of the plasma membrane fatty acid transporter protein 2 (FATP2) was measured by real-time Q-RT-PCR. (**B**) Human hepatocytes and HepG2 cells were infected with Ad-Foxa1 for 24h and were then exposed to 0.2 mM FA (oleate: palmitate, 2∶1) for an additional period lasting 16 h. The amount of non-consumed (residual) free FA, expressed as the percentage of the initial amount in the culture medium, were determined with a specific kit. (**C**) The uptake of a fluorescent FA analog (C1-BODIPY 500/510-C12) was measured in HepG2 cells and human cultured hepatocytes with an Olympus ScanR Screening Station. Data represent the mean±SD of 4–5 independent experiments. * p<0.05; ** p<0.01; *** p<0.001 in relation to Ad-Control infected cells.

We found that Foxa1-transfected cells express a 35–50% lower level of the FA transporter protein FATP2 (Fig. 5A), an abundant liver FATP and a major contributor to hepatic FA uptake [23]. We also performed functional assays to investigate whether a lower FATP2 mRNA level results in decreased FA transport. Results demonstrated that the percentage of non-consumed (residual) FA in the medium were 1.7-2.0-fold higher in cultured human hepatocytes and 1.2-1.7-fold higher in HepG2 cells, after Ad-Foxa1 transduction (Fig. 5B), which indeed suggests a lower FA uptake and usage. We wanted to confirm this finding by an alternative approach and assessed the influence of Foxa1 on the incorporation of a fluorescent FA analog (C1-BODIPY 500/510-C12) during 15 min. Results demonstrated that Ad-Foxa1-transfected HepG2 cells showed a 48–55% (human hepatocytes) and 17–31% (HepG2) decrease in FA analog uptake with respect to Ad-Control cells (Fig. 5C), thus reinforcing the idea that Foxa1 is able to reduce FA transport into hepatocytes.

**Figure 6:**
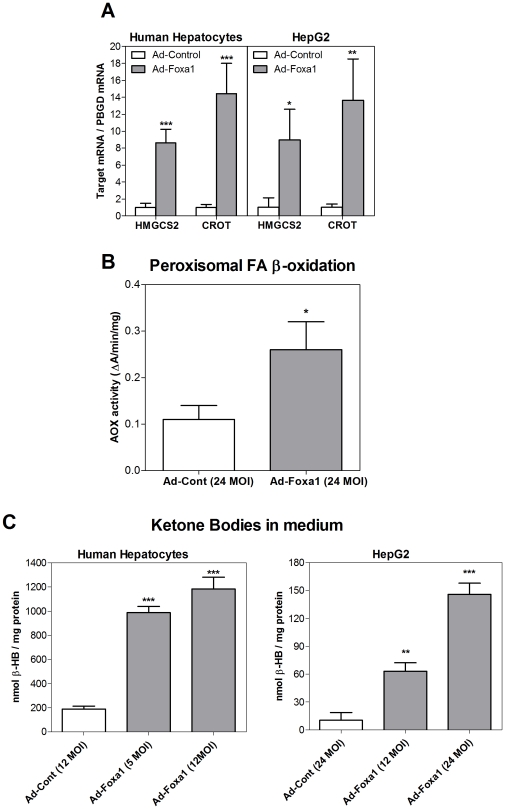
Foxa1 increases FA catabolism and KB synthesis. (**A**) Cultured cells were infected with Ad-Foxa1 or Ad-Control for 48 h, and the mRNA level of the mitochondrial 3-hydroxymethyl glutaryl coA synthase (HMGCS2, ketogenesis) and the peroxisomal carnitine O-octanoyltransferase (CROT, β-oxidation) were measured by real-time Q-RT-PCR. (**B**) HepG2 were infected with Ad-Foxa1 or Ad-Control and 48 h after infection ACOX activity was assayed spectrophotometrically in cell homogenates using palmitoyl-CoA as the substrate. Results were expressed as increments of absorbance at 502 nm (ΔA)/min/mg of protein. (**C**) Human hepatocytes and HepG2 cells were infected with Ad-Foxa1 for 24 h and were then exposed to 0.2 mM FA for an additional period lasting 24 h. The concentration of KB (β-HB, β-hydroxybutyrate) was determined in the culture medium with a specific kit. Data represent the mean±SD of 4–5 independent experiments. * p<0.05; ** p<0.01; *** p<0.001.

Next, we intended to know whether Foxa1 also regulates FA catabolism. We noted a significant up-regulation of a gene involved in peroxisomal β-oxidation (CROT: carnitine O-octanoyltransferase) in Ad-Foxa1-transfected human hepatocytes and HepG2 cells (14-fold increase, Fig. 6A). In order to know if peroxisomal β-oxidation was stimulated by Foxa1 we measured the activity of acyl-CoA oxidase (ACOX), the first and rate-limiting enzyme of this pathway. ACOX was proved to be a valid measure of peroxisomal β-oxidation in homogenates of rat liver [24]. We have assessed ACOX activity, based on H_2_O_2_-dependent dichlorofluorescein oxidation [15], [16] in cell extracts from HepG2 cells transfected with Ad-Control or Ad-Foxa1. Results demonstrate that that ACOX activity is induced 2.4 fold when cells are transfected with 24 MOI of Ad-Foxa1 (Fig. 6B) and indicate that Foxa1 is able to stimulate peroxisomal FA β-oxidation.

In the context of FA catabolism, we have also found that Foxa1 activates ketogenesis through the induction of the mitochondrial 3-hydroxymethyl glutaryl-CoA synthase (HMGCS2), the rate-limiting enzyme of this pathway. HMGCS2 mRNA was induced 9-fold (Fig. 6A), and the level of ketone bodies (KB) increased 5.2-6.3-fold in the culture medium of Ad-Foxa1-infected human hepatocytes and 6.1-14.3 fold in HepG2 cells (Fig. 6C). Thus, our results demonstrate that peroxisomal FA β-oxidation and catabolism toward KB is enhanced by Foxa1.

**Figure 7:**
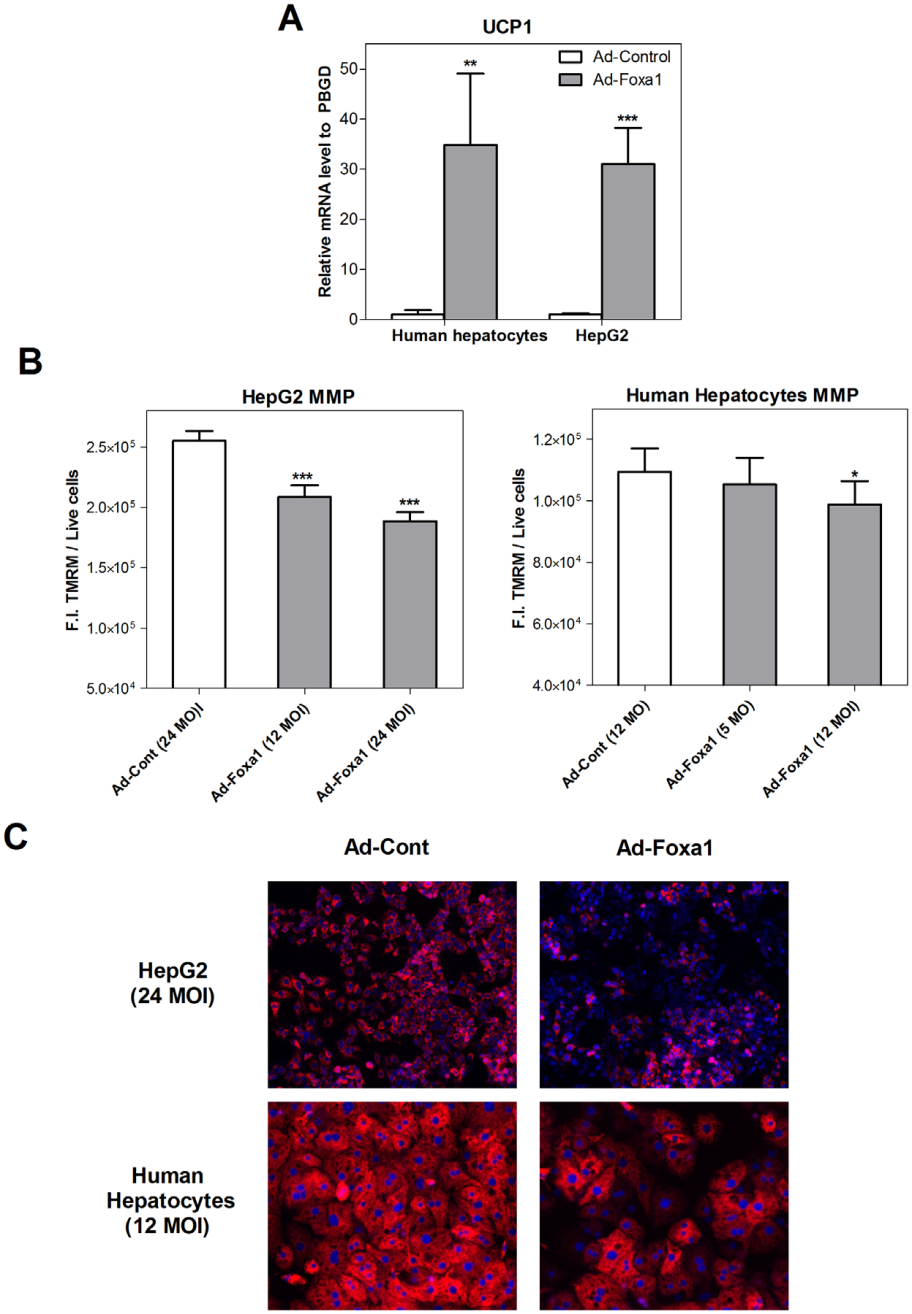
Foxa1 up-regulates UCP1 and decreases MMP. Cultured cells were infected with Ad-Foxa1 or Ad-Control (48 h), the mRNA level of the mitochondrial uncoupling protein 1 (UCP1) was measured by real-time Q-RT-PCR (**A**), and the Mitochondrial Membrane Potential (MMP) was measured using the TMRM probe in an Olympus Sca
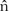
R Screening Station (**B**). Data represent the mean±SD of 4–5 independent experiments. ** p<0.01; *** p<0.001. (**C**) Representative microphotographs of HepG2 cells and human cultured hepatocytes after 48 h of adenoviral transfection. Cells were stained with TMRM (red) for mitochondrial membrane potential and Hoechst 33342 (blue) for nuclei.

Uncoupling protein 1 (UCP1) is not a gene with a constitutive expression in the liver and, consequently, UCP1 mRNA expression was very low in control human hepatocytes (cycle threshold >34). However, UCP1 was induced by Foxa1 up to a significant mRNA level (30-35-fold increase, Fig. 7A). If UCP1 function increases, mitochondria should be partially uncoupled and the MMP reduced [25]. We measured the MMP in living cells by using the Olympus Sca
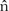
R Screening Station and the fluorescent probe TMRM to find that Ad-Foxa1-infected cells had a 20–25% reduction in HepG2 and a 4–10% decrease in human hepatocytes (Fig. 7B). Microphotographs in Fig. 7C show the decrease in red fluorescence (MMP) when cells are infected with Ad-Foxa1. These results suggest that Foxa1 can stimulate the leakage of protons into the mitochondrial matrix to dissipate energy rather than allowing it to be captured in ATP.

### Foxa1 expression decreases in human NAFL

A transcription factor that is able to coordinate a metabolic response should be subjected to modulation by metabolic signals and mediators. Foxa1 is not phosphorylated and inhibited by insulin-signaling cascades. We investigated other possible metabolic scenarios and found that the expression of this factor is down-regulated in human NAFL.

**Figure 8:**
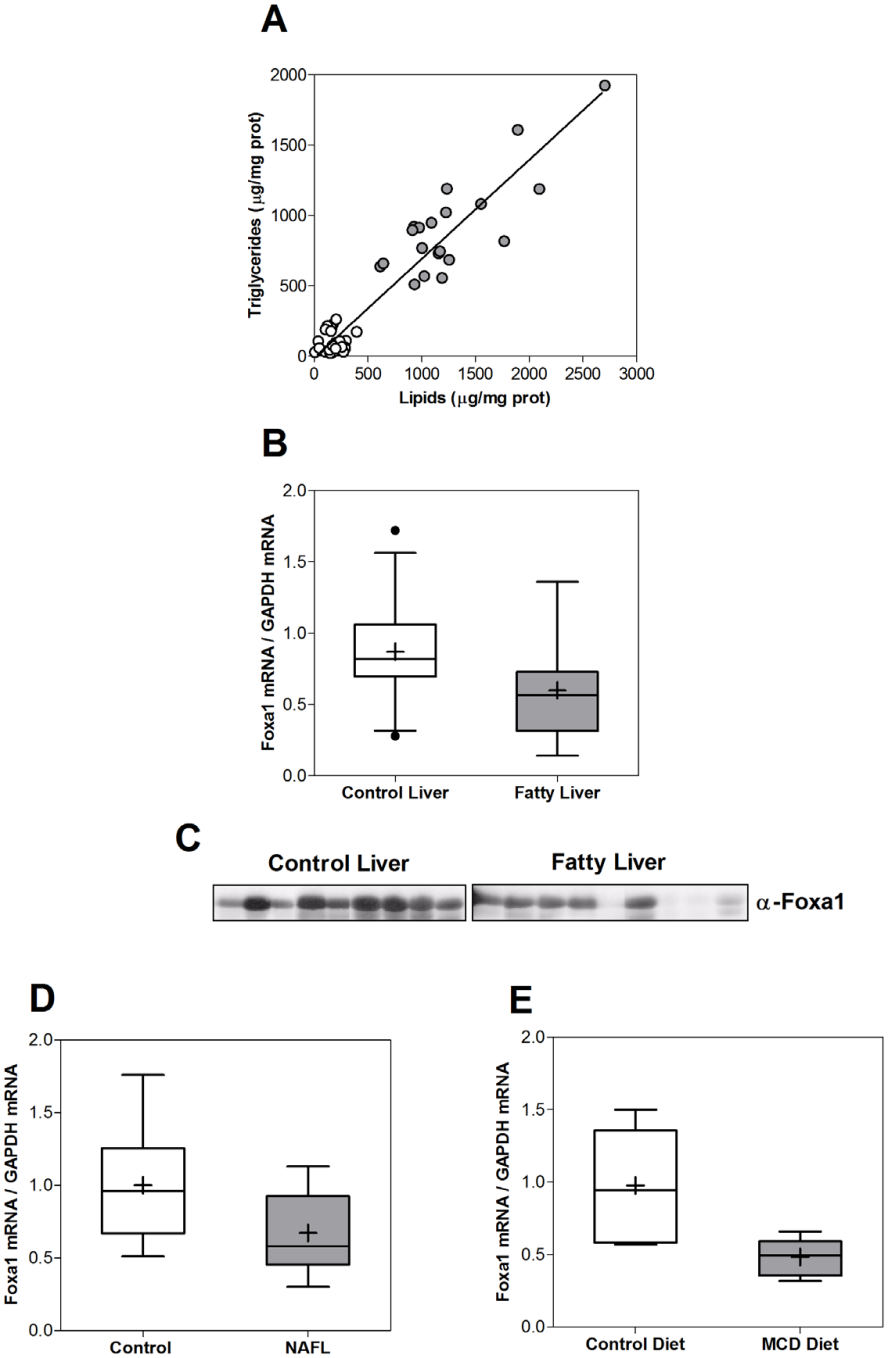
Foxa1 expression is reduced in human and rat fatty liver. (**A**) Liver samples (100 mg) from the Human Liver Bank HLaFe-CIBERehd were homogenized, extracted with methanol-chloroform and evaporated under nitrogen. Total lipids and TG were measured in the lipid residue using colorimetric kits and livers were classified as fatty livers (closed circles) when total lipids were >600 µg/mg prot and TG>500 µg/mg prot (n = 20), or as nonsteatotic livers (open circles) when total lipids were <400 µg/mg prot and TAG<300 µg/mg prot (n = 30). (**B**) Total RNA was purified from matching Liver Bank samples and the mRNA level of Foxa1 was determined by real-time Q-RT-PCR analysis. (**C**) Protein extracted with T-PER from representative liver homogenates was analyzed by immunoblotting with a monoclonal antibody against human Foxa1 (sc-101058). (**D**) Total RNA was also purified from NAFL (n = 17) and cholelithiasis (n = 17) patients' liver biopsies and the mRNA level of Foxa1 was quantified by real-time Q-RT-PCR. (**E**) Adult male OFA rats were randomized into two experimental groups (n = 6 per group) and fed with either MCD or a standard isocaloric diet for 7 weeks *ad libitum*. Total RNA was purified and the expression level of Foxa1 determined as explained above. Box plots represent the 25^th^, the median and the 75^th^ percentiles, a cross depicts the mean, whiskers indicate the 95^th^ and 5^th^ percentile, and dots represent outliers. GAPDH mRNA was used for normalization.

We analyzed the expression of Foxa1 in a collection of human livers from the Human Liver Bank HLaFe-CIBERehd, which were classified as steatotic or nonsteatotic according to their lipid content (Fig. 8A, see donors' characteristics in Table 1 - left). We measured the level of Foxa1 mRNA by Q-RT-PCR, and found a significantly lower level in steatotic livers (68.8%, p<0.01) (Fig. 8B). This down-regulation was also observed at the protein level, as determined by immunoblotting of human Foxa1 in representative control and fatty livers (Fig. 8C). To confirm these findings we also measured Foxa1 mRNA levels in patients subjected to liver needle biopsy (see patients' characteristics in Table 1 - right). In agreement with our previous results, we discovered lower Foxa1 mRNA levels in the group of patients with NAFL (67.1%, p<0.01) (Fig. 8D).

**Table 1:** Anthropometric and analytical parameters of steatotic and non-steatotic liver donors and patients.

	Human Liver Bank^1^ (Mean±SD)		Human Liver Biopsies^2^ (Mean±SD)	
	Non-Steatotic (n = 30; 14m 16f)	Steatotic (n = 20; 14m 6f)	Non-Steatotic (n = 17; 2m 15f)	Steatotic (n = 17; 8m 9f)
Age (years)	60±18	47±15*	50±10	50±13
BMI (kg/m^2^)	27±3	30±5*	27±4	30±3*
**Blood Tests**				
γGT (IU/L)	56±69	56±45	25±17	37±22
AST (IU/L)	44±37	45±25	17±4	22±7*
ALT (IU/L)	33±27	38±30	16±5	28±18*
ALP (U/L)	91±42	103±70	72±26	75±27
Quick's value (%)	69±23	80±16	ND	ND
Bilirubin total (mg/dL)	1.0±1.0	1.2±1.7	ND	ND
Creatinine (mg/dL)	1.2±1.2	1.3±1.5	ND	ND
Urea (mg/dL)	39±19	33±10	ND	ND
Glucose (mg/dL)	178±50	191±89	90±10	97±14
Hemoglobin (g/dL)	16±20	19±26	ND	ND
TG (mg/dL)	ND	ND	127±50	161±84
Chol-Tot (mg/dL)	ND	ND	200±35	211±44
Chol-HDL (mg/dL)	ND	ND	47±10	47±13
Insulin (µU/L)	ND	ND	6.2±2.7	10.7±6.3*
HOMA-IR	ND	ND	0.8±0.3	1.4±0.8*
**Liver Tests**				
Tot Lip (µg/mg prot)	183±81	1269±509***	ND	ND
TG (µg/mg prot)	93±70	919±354***	ND	ND

1 Liver donors showed normal analytical blood test and, apart of the hepatic lipid concentration and the BMI, no major differences were found between steatotic and non-steatotic.

2 Patients with NAFL (grade 1 or 2) showed a higher BMI, a slight elevation of transaminases and a statistically higher insulin level and HOMA (Homeostasis Model Assessment) index.

*p<0.05;

***p<0.001; m, male; f, female; ND, not determined.

Finally, we decided to know if the steatosis-induced repression of Foxa1 occurs in NAFL animal models. Adult male OFA rats were fed with either a methionine and choline deficient (MCD) diet or standard isocaloric chow. The level of Foxa1 in steatotic rat livers dropped to 50% (p = 0.013) (Fig. 8E). Our results demonstrate that human and rat NAFL is associated with a decrease in Foxa1 expression.

### Foxa1 repression in human NAFL is likely mediated by higher PKC activity/expression

**Figure 9:**
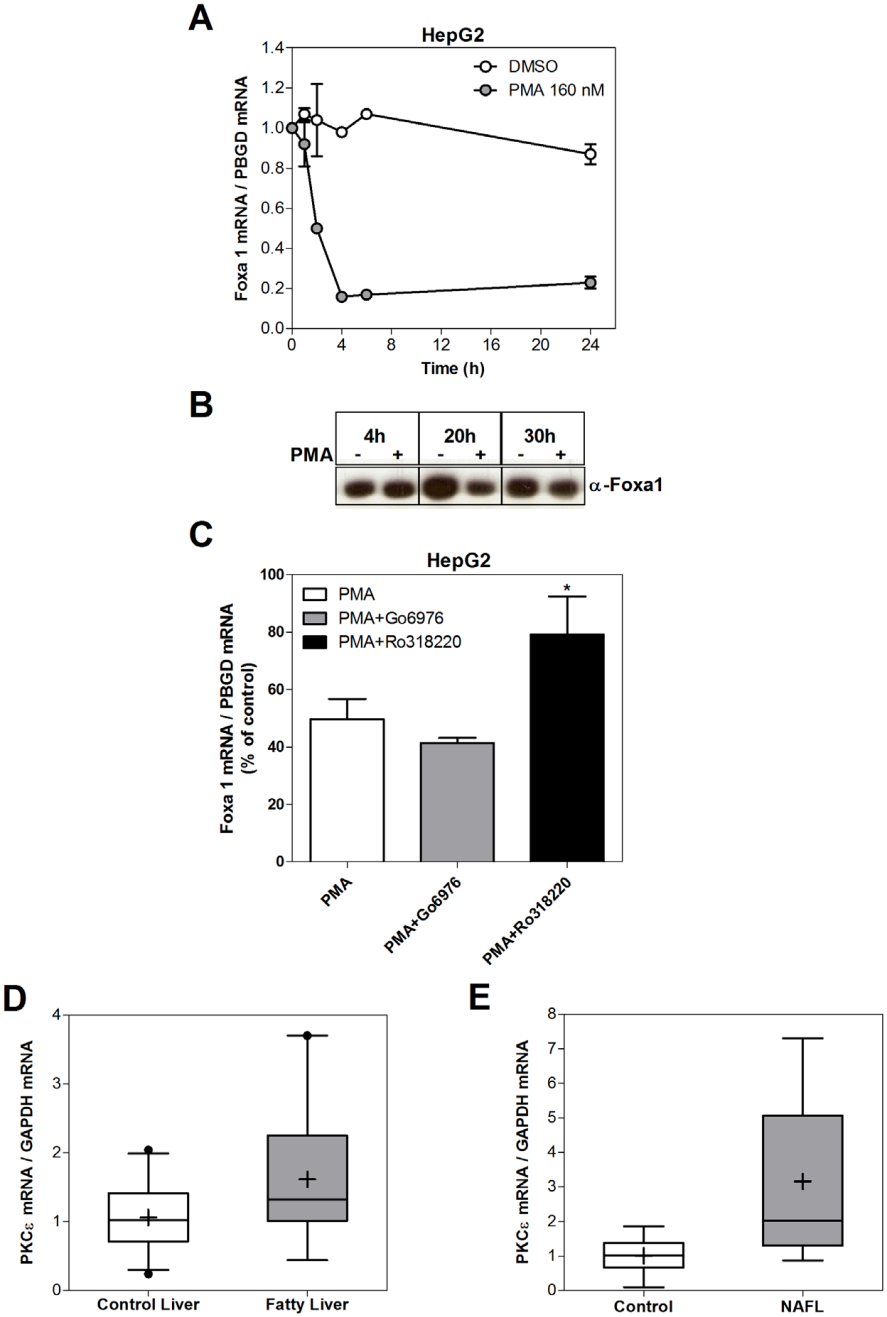
Foxa1 repression in NAFL correlates with higher PKC activity/expression. (**A**) HepG2 cells were exposed to the PKC activator PMA (phorbol 12-myristate 13-acetate), and the level of Foxa1 mRNA was measured at different time points. Data represent the mean±SD of 3 independent experiments. (**B**) Foxa1 protein level in HepG2 cells was determined by immunoblotting (sc-101058 antibody) after 4, 20 and 30 h of exposure to PMA (+) or DMSO (−). (**C**) HepG2 cells were pretreated with PKC inhibitors, Gö-6976 (200 nM) or Ro-318220 (1 µM), for 1 h, and thereafter exposed to 160 nM PMA. Two hours latter cells were collected and Foxa1 mRNA level was determined by Q-RT-PCR. PBGD mRNA was used for normalization. Data represent the percentage of Foxa1 in treated samples (PMA or PMA plus PKC inhibitors) respect to control samples (DMSO or inhibitors alone). (**D–E**) Protein kinase C epsilon (PKCε) mRNA level was determined by real-time Q-RT-PCR analysis in the human liver samples described in the legend of Fig. 8B and 8D.

The down-regulation of Foxa1 in steatotic liver could result from different signaling pathways and mechanisms. One possibility is hyperinsulinemia. NAFL favors insulin resistance and high insulin plasma levels could cause Foxa1 down-regulation. However, we did not observe Foxa1 down-regulation when human hepatocytes and HepG2 cells were exposed to increasing concentrations of insulin (data not shown). Other possibility is that fat accumulation triggers intracellular signals or mediators that cause Foxa1 down-regulation. Excessive FA delivery into hepatocytes results in accumulation of intermediate lipid metabolites such as diacylglycerol, which can activate protein kinase C (PKC) that, in turn, binds to the insulin receptor and inhibits its tyrosine kinase activity leading to hepatic insulin resistance. We wanted to know if the PKC signaling pathway could also lead to Foxa1 gene repression. To assess this possibility, we exposed cultured cells to the PKC activator phorbol 12-myristate 13-acetate (PMA) and measured the level of Foxa1 mRNA (Fig. 9A) and protein (Fig. 9B). The data obtained demonstrated that PMA represses Foxa1 expression in HepG2 cells.

In order to prove that the effect of PMA was mediated by PKC activation, isoform specific PKC inhibitors were used. We found that the general PKC-inhibitor Ro318220 was able to counteract the repressive effect of PMA. However, Gö-6976, a more specific inhibitor of conventional PKCs (Ca^2+^-dependent: alpha, beta I/II and gamma), did not affect the response of Foxa1 to PMA (Fig. 9C). This points to the novel PKCs (Ca^2+^-independent: delta, epsilon, theta and nano) as the likely isoforms mediating the down-regulation of Foxa1. We did not observe the repression of Foxa1 by PMA in human hepatocytes (n = 2), where hepatocyte isolation, culture conditions, medium composition and donor characteristics may negatively influence the activation of PKC by PMA.

On the other hand, we found that the novel protein kinase C epsilon (PKCε) mRNA was more abundant in human fatty livers (153%, p<0.05) (Fig. 9D) and in NAFL patients (316%, p<0.01) (Fig. 9E), suggesting that higher PKC expression and/or activity (likely PKCε) could be associated with the down-regulation of Foxa1 in human steatotic livers.

## Discussion

No gross abnormalities have been observed in the livers of Foxa-1, -2 or -3 null mice [9], [11], [12]. The three Foxas share a great homology in the DNA-binding domain and in the recognition sequences, and can compete for the same binding sites [26]. Consequently, it has been suggested that Foxa factors can compensate each other, which hinders the use of loss-of-function strategies. To circumvent this functional redundancy, transgenic and adenovirus-infected mice have been generated [3], [27], [28], [29]. However, most of these studies have been addressed to elucidate the role of Foxa2 in bile acid [27], glucose [27], [29] and lipid [3], [28] metabolism. Moreover, very few studies have attempted to corroborate and determine the relevance of Foxa factors in the human liver.

In the present study, we have investigated the regulation of Foxa1 in human NAFL, and attempted to uncover the relevance of this transcription factor in human hepatocytes and HepG2 by gain-of-function experiments. Cell transfection with an adenoviral vector encoding Foxa1 compensated for the decreased Foxa1 mRNA expression observed in cultured hepatocytes and HepG2 cells but also exceeded the level found in human liver. A potential limitation of these overexpression studies is that mechanisms that normally regulate gene transcription might be forced or overwhelmed. Nevertheless, all our experimental evidence strongly suggest that Foxa1 plays a key role in the control of lipid metabolism in the human liver, which has not been previously anticipated in animal models.

One important observation is the capacity of Foxa1 to simultaneously inhibit the expression of most of the genes involved in hepatic TG and VLDL synthesis pathways. Previous findings demonstrated that elevated levels of Foxa2 activate the expression of lipogenic genes (inducing steatosis) [28] and MTP (increasing VLDL secretion) [4]. Our results demonstrate that Foxa1 can activate the opposite response, which leads to decreased intracellular lipids and ApoB100 secretion. The negative effect of Foxa1 on DGAT2 is of particular relevance as knocking down DGAT2 protects against fat-induced hepatic steatosis and insulin resistance [30], [31].

A second important and novel observation is the capacity of Foxa1 to inhibit FA uptake by human hepatocytes. Our results also suggest that this could be due to the repression of FATP2 by Foxa1. FATP2 and FATP5 are the two major FATPs in the liver. FATP2 knockdown by shRNA reduces FA uptake and hepatosteatosis in the face of continued high-fat feeding [23]. Previous results with other Foxa members have not shown any inhibition on FA transporters, instead an activating effect of Foxa2 on the FA translocase FAT/CD36 has been reported [3].

A third important finding is the capacity of Foxa1 to activate FA catabolism and increase energy expenditure. We observed a Foxa1-dependent increase in CROT (peroxisomal β-oxidation) and HMGCS2 (ketogenesis) mRNA levels, and in peroxisomal β-oxidation and extracellular KB, which demonstrates enhanced FA degradation. This observation is in line with previous findings demonstrating that Foxa2 activates lipid catabolism and ketogenesis in the liver through the activation of a number of genes, including HMGCS2 and CROT [3], [7]. The considerable induction of UCP1 by Foxa1 in human hepatocytes could also contribute to its antisteatotic effect. UCP1 uncouples oxidative phosphorylation and promotes the dissipation of cellular biochemical energy. Forced UCP1 expression in the liver inhibits FA synthesis with a concomitant enhancement of FA oxidation, resulting in less fat in the liver [32]. We have shown that endogenous UCP1 mRNA expression can be activated by Foxa1 in human hepatocytes, and that this associates with a lower MMP potential which suggests a new mechanism to dissipate energy and to reduce fat in the liver.

The molecular mechanism by which Foxa1 triggers the transcriptional responses observed in this study remains to be elucidated. However, the mechanism of repression by Foxa1 has been attributed to an antagonistic interplay between Foxa1 and Foxa2 [33]. It has been shown that both Foxa1 and Foxa2 are activators of gene expression, but in the context of native chromatin, Foxa1 is a poorer activator than Foxa2. Foxa1 does not appear to act as a classic biochemical repressor but rather exerts its negative effect by competing for the same cis-acting promoter elements with the more efficient activator Foxa2 [33]. In this context, we have observed that Foxa2 mRNA was down-regulated by Ad-Foxa1 in human hepatocytes and HepG2 cells. A lower Foxa2 expression along with the adenovirus-mediated upregulation of Foxa1 can lead to an efficient exchange of Foxa2 by Foxa1 in the promoters of many lipid metabolism target genes, leading to lower transcriptional activity.

Other major difference between Foxa1 and 2 is that plasma insulin selectively inhibits Foxa2 by phosphorylation at Thr 156 [3], [6], [7], which is absent in the Foxa1 protein. Consequently, Foxa2 is inactive in the fed state and in insulin-resistant models [3], whereas Foxa1 is constitutively active. Thus, the role of Foxa1 could be of particular relevance under both the fed and insulin-resistant conditions, where Foxa2 remains inactive.

Moreover, our results also suggest that, in the human liver, Foxa1 could be more abundant than Foxa2 and 3. Our results are based on absolute quantitative RT-PCR with DNA standards. We used DNA standards because they have larger quantification range and greater sensitivity, reproducibility, and stability than RNA standards [34]. However, potential differences in the efficiency of the reverse transcription for each Foxa mRNA are not taken into account with this experimental approach. Therefore, despite our results suggest that Foxa1 is a major form of the Foxa family in human liver, this needs to be confirmed with alternative approaches at both mRNA and protein level.

NAFL is very frequently associated with obesity and the metabolic syndrome, and has been suggested to be a key triggering factor for systemic insulin resistance [35]. High levels of a constitutively active Foxa2 protein (Foxa2T156A), but not the wild-type protein, can decrease liver steatosis [3], suggesting that Foxa2 could be a pharmacological target for the treatment of NAFL and for improving liver insulin sensitivity [36]. However, other studies have shown that high levels of Foxa2 in transgenic mice cause postnatal steatosis [28], which likely results from *de novo* lipid synthesis. In the present study, we found that high levels of Foxa1 reduce steatosis in human hepatocytes and hepatoma cells. Thus, Foxa1 appears to be a novel target for the therapy and prevention of human NAFL.

The expression analysis performed in the different human sample groups and in rats has revealed that Foxa1 is down-regulated in NAFL. This fact, along with the findings supporting an antisteatotic role of Foxa1, led us to hypothesize that the down-regulation of Foxa1 in NAFL is likely to increase uptake and storage of excessive circulating FA into hepatocytes. Elucidating the mechanism of this down-regulation could enable us to know how to counteract this response and reduce steatosis. In this sense, high-fat diet mice accumulate more liver diacylglycerol than wild-type mice, which causes insulin resistance by activating PKCε, a specific isoform of PKC [37], [38]. Our data demonstrates that PKC activation also represses Foxa1 in HepG2 cells. The use of specific PKC inhibitors points to the novel PKCs as the likely isoforms involved. Moreover, we demonstrate that human NAFLs have an increased PKCε mRNA expression. This evidence supports the involvement of novel PKCs in Foxa1 down-regulation, but the specific isoform and pathway remains to be determined. Blocking this specific PKC could prevent the down-regulation of Foxa1 in human NAFL which, in turn, could help not only reduce intracellular lipids, but alleviate insulin resistance in humans.

How does the activity of a kinase decrease expression of a gene? In the regulatory systems for other genes, *de novo* synthesis of repressors (inhibited by cycloheximide) is proposed to be the mechanism of down-regulation by PKC [39]. In other studies, a more direct effect of PKC on the phosphorylation of a repressor has been suggested [40].

We conclude that Foxa1 can act coordinately in different pathways to reduce hepatic lipid accumulation by inhibiting FA transport and TG synthesis while stimulating FA catabolism and dissipating surplus energy via enhanced UCP1 expression. Moreover as Foxa1 is repressed in NAFL, its pharmacological activation could counteract lipid deposition, and could prove useful for the therapy and prevention of NAFLD and insulin resistance.

## Supporting Information

Table S1 **List of primers used for quantitative RT-PCR.**
(PDF)

Table S2 **Microarray expression analysis of lipid and carbohydrate metabolism genes altered by Foxa1 in human liver cells.**
(PDF)
