# Supplementary material for: Foxa1 Reduces Lipid Accumulation in Human Hepatocytes and Is Down-Regulated in Nonalcoholic Fatty Liver
Source: PLoS One. 2012 Jan 6;7(1):e30014. doi: 10.1371/journal.pone.0030014 (PMC3253125; doi:10.1371/journal.pone.0030014)
Supplement: Table S1 — List of primers used for quantitative RT-PCR. (PDF) [file pone.0030014.s001.pdf]

Table S1: ***Primers used for quantitative RT-PCR***

Primer	Forward (5'->3')	Reverse (5'->3')	Nucleotide position
ACAT2	TGGCTCAACGCCTTTGCCGA	CAAGGAGCCGCAGCCCATCC	1172-1331
APO B	TGGATTCCACATGCAGCTCAACCG	TGAGGTGCGCTTTTCCTTCCCAT	11866-12057
CROT	CCTTGAATCAGTGAAACCATTTGC	GCTGCAGGACCCGCAAAGTTGA	350-568
DGAT2	TGGGGGCTGGTGCCCTACTC	AATTGGCCCCGAAGGCTGGC	1232-1446
FATP2	TGCCCTTTTACCACAGTGCTGC	AGCCATTTCCCAGTGCCAGTCT	1023-1253
FOXA 1 pair1	TGGGGGGTTTGTCTGGCATA	ATTGGTTTGGGGTTGTCTTTG	1742-1962
FOXA 1 pair2	CTCTAGGCAGCGCCTCGGTGA	CAGCATGGCTATGCCAGACAAACCC	1625-1770
FOXA 2	CACCACCACCAACCCCACAAA	TGCAACACCGTCTCCCCAAAG	1380-1675
FOXA 3	GGACGCGCCCTACAACTTC	TTAAGCAAAGAGCGGGAATAG A	1079-1240
FOXA1 rat	CCAGGGTGGCTCCAGGATGTT	CTAAGCCCGGGTTGGCGTAGG	42-277
GAPDH	ATGCTGGCGCTGAGTACGTC	GGGCAGAGATGATGACCCTT	368-472
GAPDH rat	AGGGCTCATGACCACAGTCCAT	GCCAGTGAGCTTCCCGTTCAG	510-684
GPAT1	GGCCTACAGCTCTGCTGCCA	GCATTGAGGTAGAAAAGTGCTGCTCA	2388-2637
HMGCS2	GCAGCATCGCCGAAAGTA	CCTAGTCCATAGCACCATAAGCC	1453-1716
MTP	TGTGGTATCGTGAGTCTAAAACCCGA	TGCCTGTGGACAGCCTTTCGT	2562-2807
PBGD	CGGAAGAAAACAGCCCAAAGA	TGAAGCCAGGAGGAAGCACAGT	189-482
PKCε	CTCCCAGCGGTTCAGCGTCA	GACAAGGCTTTCCGAATGTT	896-1300
UCP1	ACAACCGAAGGCTTGACGGGT	GCCGACACCAAGTGGCAGGG	572-732
