# Supplementary material for: Foxa1 Reduces Lipid Accumulation in Human Hepatocytes and Is Down-Regulated in Nonalcoholic Fatty Liver
Source: PLoS One. 2012 Jan 6;7(1):e30014. doi: 10.1371/journal.pone.0030014 (PMC3253125; doi:10.1371/journal.pone.0030014)
Supplement: Table S2 — Microarray expression analysis of lipid and carbohydrate metabolism genes altered by Foxa1 in human liver cells. (PDF) [file pone.0030014.s002.pdf]

Table S2. ***Microarray expression analysis of lipid and carbohydrate metabolism genes altered by Foxa1 in human liver cells***

				Cultured Human Hepatocytes			Human Hepatoma HepG2		
**Function**	**Symbol**	**Gene name (ALIASES)**	**Acc Number**	**Fold Change**	**FDR**	**moderated pvalue**	**Fold Change**	**FDR**	**moderated pvalue**
**Extracellular hydrolysis of TG and lipoprotein uptake**	LIPC	lipase, hepatic (LIPH, HL)	NM_000236	-3.5	0.022	0.001	-3.8	0.000	0.000
	LIPG	lipase, endothelial (EL, EDL)	NM_006033	-1.8	0.371	0.096	-1.3	0.161	0.070
	VLDLR	very low density lipoprotein receptor	NM_003383	-1.8	0.057	0.007			
**FA uptake and vectorial acylation**	SLC27A2	solute carrier family 27 (fatty acid transporter), member 2 (FATP2)	NM_003645	-3.9	0.008	0.000	-2.1	0.000	0.000
	CAV1	caveolin 1, caveolae protein, 22kDa	NM_001753	-1.9	0.136	0.028			
	ACSL5	acyl-CoA synthetase long-chain family member 5 (ACS2)	NM_016234	-2.6	0.003	0.000	-1.9	0.000	0.000
	ACSM5	acyl-CoA synthetase medium-chain family member 5	NM_017888	-2.2	0.004	0.000			
	ACSM2B	acyl-CoA synthetase medium-chain family member 2B	NM_182617				-1.9	0.000	0.000
	ACSM3	acyl-CoA synthetase medium-chain family member 3	NM_005622				-2.0	0.000	0.000
	FABP1	fatty acid binding protein 1, liver	NM_001443	-1.7	0.150	0.033			
	FABP2	fatty acid binding protein 2, intestinal	NM_000134				-1.5	0.045	0.014
	FABP5	fatty acid binding protein 5	NM_001444				-2.0	0.000	0.000
**TG synthesis**	GPAT1	glycerol-3-phosphate acyltransferase (GPAM)	NM_020918	-2.9	0.093	0.015	-2.4	0.000	0.000
	AGPAT5	1-acylglycerol-3-phosphate O-acyltransferase 5	NM_018361	-2.5	0.002	0.000			
	AGPAT9	1-acylglycerol-3-phosphate O-acyltransferase 9	NM_032717	-2.8	0.019	0.001			
	AGPAT7	1-acylglycerol-3-phosphate O-acyltransferase 7	NM_153613				-1.4	0.045	0.014
	LPIN2	lipin 2	NM_014646	-1.9	0.139	0.029			
	LPIN1	lipin 1	NM_145693	1.5	0.188	0.047			
	PPAP2A	phosphatidic acid phosphatase type 2A (PAP2)	NM_003711	-1.9	0.034	0.003			
	PPAP2B	phosphatidic acid phosphatase type 2B	NM_003713				-1.5	0.000	0.000
	DGAT2	diacylglycerol O-acyltransferase homolog 2	NM_032564	-2.1	0.021	0.001	-2.3	0.000	0.000
	DGAT1	diacylglycerol O-acyltransferase homolog 1	NM_012079				-1.5	0.000	0.000
	MOGAT2	monoacylglycerol O-acyltransferase 2	NM_025098	-1.8	0.433	0.181	-1.5	0.000	0.000

**Lipid droplet (structure, formation and hydrolases)**	PNPLA3	patatin-like phospholipase domain containing 3 (adiponutrin)	NM_025225	-2.0	0.032	0.003	-1.9	0.000	0.000
	ADFP	adipose differentiation-related protein-perilipin 2 (adipophilin)	NM_001122	-1.6	0.260	0.079	-1.7	0.000	0.000
	CIDEC	cell death-inducing DFFA-like effector c	NM_022094	-2.6	0.062	0.007	-1.5	0.000	0.000
	CIDEB	cell death-inducing DFFA-like effector b	NM_014430				-1.4	0.000	0.000
**VLDL synthesis**	SOAT2	acyl coenzyme A:cholesterol acyltransferase 2 (ACAT2)	NM_003578	-1.8	0.051	0.005	-2.5	0.000	0.000
	MTTP	microsomal triglyceride transfer protein (MTP)	NM_000253	-4.9	0.045	0.004	-1.9	0.000	0.000
	APOB	apolipoprotein B (ApoB48, ApoB100)	NM_000384	-4.4	0.020	0.001	-1.3	0.002	0.000
	APOC3	apolipoprotein C-III	NM_000040				-3.2	0.000	0.000
**FA synthesis, elongation and desaturation**	ACACB	acetyl-Coenzyme A carboxylase beta (ACC2)	NM_001093	-1.8	0.145	0.031			
	ACACA	acetyl-Coenzyme A carboxylase alpha (ACC1)	NM_198839	-1.5	0.150	0.032			
	FASN	fatty acid synthase	NM_004104	-1.4	0.479	0.218	-1.5	0.039	0.011
	ELOVL6	ELOVL family member 6, elongation of long chain fatty acids	NM_024090	-3.7	0.002	0.000			
	ELOVL5	ELOVL family member 5, elongation of long chain fatty acids	NM_021814	-1.6	0.100	0.017			
	ELOVL7	ELOVL family member 7, elongation of long chain fatty acids	NM_024930	1.7	0.599	0.330	6.1	0.000	0.000
	SCD	stearoyl-CoA desaturase (delta-9-desaturase) (SCD1)	NM_005063	-1.5	0.437	0.185	-1.4	0.011	0.002
	ACOX3	acyl-Coenzyme A oxidase 3, pristanoyl	NM_003501	-1.7	0.035	0.003			
	FADS2	fatty acid desaturase 2 (D6D)	NM_004265	-2.1	0.136	0.027	-1.4	0.028	0.008
	FADS3	fatty acid desaturase 3	NM_021727	-2.5	0.003	0.000	-1.2	0.002	0.000
	FADS1	fatty acid desaturase 1 (D5D)	NM_013402	-3.0	0.024	0.001	-1.3	0.005	0.001
**FA oxidation and ketone body synthesis**	HMGCS2	3-hydroxy-3-methylglutaryl-Coenzyme A synthase 2	NM_005518	4.1	0.056	0.002	2.0	0.002	0.000
	CROT	carnitine O-octanoyltransferase	NM_021151	9.5	0.004	0.000	5.6	0.000	0.000
	ACADSB	acyl-Coenzyme A dehydrogenase, short/branched chain	NM_001609	6.4	0.001	0.000	2.3	0.000	0.000
**Carbohydrate metabolism**	G6PC	glucose-6-phosphatase, catalytic subunit	NM_000151	46.9	0.001	0.000	1.3	0.050	0.016
	PDK2	pyruvate dehydrogenase kinase, isozyme 2	NM_002611	2.1	0.041	0.004			
	PDK4	pyruvate dehydrogenase kinase, isozyme 4	NM_002612	2.0	0.444	0.144			
	GYS2	glycogen synthase 2 (liver)	NM_021957	-8.1	0.002	0.000			
	PKLR	pyruvate kinase, liver and RBC	NM_000298	-1.4	0.609	0.340	-1.8	0.000	0.000
